# Another Death from Chloroform

**Published:** 1866-07

**Authors:** 


Another Death from Chloroform.—A few days since another victim to this murderous anæsthetic died in a dentist's chair in the city of Philadelphia. An inquest was held upon the body, from the report of which, as we find it in the newspapers, we make the following extract:

"The evidence at the inquest was short, and unusually to the point. It was as follows:

"Mrs. Siemmers sworn.—I live at 1142 South Eighth street; I have known Mrs. Lyster several years; she called at my house requesting me to accompany her to Mr. Slack's, the dentist; she requested me because her sister was very nervous; this was a week ago; she asked Dr. Slack to give her chloroform; he said that he didn't use chloroform, and recommended nitrous oxide gas; she said she had taken chloroform before, and preferred it to anything else; an appointment was made for Monday at 2 o'clock. Witness accompanied Mrs. Lyster according to appointment; the doctor fitted the forceps to her teeth; Mrs. Lyster was extremely nervous; he put a cork between her teeth; he said that this was necessary; he then placed a cloth over her nostrils and dropped chloroform upon it; in a minute or two she turned deathly white and said she was very sick; she didn't speak afterwards, but went into convulsions and died soon; Mrs Slack came in, and we all rubbed her and tried to restore her, but it was of no use

Dr. Wm. W. Slack, sworn—Testified that Mrs. Lyster called upon him a week ago to have some teeth extracted; she seemed exceedingly nervous and apprehensive of pain; she asked how pain could be avoided; witness suggested that she should take nitrous oxide; she said that she had taken chloroform before, and preferred that; an appointment was made accordingly, though witness refused to administer more than enough to paralyze the nerve—not to produce unconsciousness; this was after she assured me that she had no affection of the heart, nor any complaint that could be prejudiced by its use; bought the chloroform on Sadurday night; I gave to a little child on the same day a larger inhalation than that which Mrs. Lyster took.

Dr. Shapleigh testified that he had made a *post-mortem* upon the deceased. Every internal organ was perfectly healthy. There was congestion of the lungs and bronchial tubes. This may or may not have been the result of chloroform. — This closed the testimony. The verdict was a simple one — that

Mrs. Lyster died from the effects of chloroform, administered as an anæsthetic by Dr. Slack, in a very moderate quantity; that the deceased requested its application to allay the pain consequent upon the extraction of her teeth.

